# HIV treatment simplification to elvitegravir/cobicistat/emtricitabine/tenofovir disproxil fumarate (E/C/F/TDF) plus darunavir: a pharmacokinetic study

**DOI:** 10.1186/s12981-017-0185-4

**Published:** 2017-11-02

**Authors:** Marianne Harris, Bruce Ganase, Birgit Watson, P. Richard Harrigan, Julio S. G. Montaner, Mark W. Hull

**Affiliations:** 1 British Columbia Centre for Excellence in HIV/AIDS, Vancouver, BC Canada; 2 AIDS Research Program, St. Paul’s Hospital, Vancouver, BC Canada; 3 Department of Family Practice, Faculty of Medicine, University of British Columbia, Vancouver, BC Canada; 4 Division of AIDS, Faculty of Medicine, University of British Columbia, Vancouver, BC Canada

**Keywords:** HIV, Antiretrovirals, Elvitegravir, Cobicistat, Darunavir

## Abstract

**Background:**

As a simplification strategy for treatment-experienced HIV-infected patients who have achieved virologic suppression on a multi-drug, multi-class antiretroviral regimen, the aim of this study was to evaluate the safety, efficacy, and pharmacokinetics of once-daily elvitegravir/cobicistat/emtricitabine/tenofovir disproxil fumarate (E/C/F/TDF) with darunavir.

**Methods:**

A single arm, open-label 48-week study was conducted of regimen simplification to E/C/F/TDF plus darunavir 800 mg daily from stable therapy including two nucleoside/nucleotide reverse transcriptase inhibitors, a ritonavir-boosted protease inhibitor, and an integrase inhibitor. Participants had plasma HIV viral load consistently < 200 copies/mL for ≥ 6 months, estimated glomerular filtration rate (eGFR) ≥ 60 mL/min, and no genotypic resistance to major components of the study regimen. Plasma viral load was measured at weeks 2 and 4, then every 4 weeks throughout the study. Safety laboratory assessments were conducted at baseline and at weeks 12, 24, 36, and 48. Antiretroviral drug concentrations were measured at baseline and once ≥ 2 weeks after the regimen change.

**Results:**

Ten HIV-infected adults (8 male and 2 female; median age 50.5 years) were enrolled. All maintained virologic suppression on the new regimen for 48 weeks. One subject experienced a decrease in eGFR from 62 mL/min at baseline to 52 mL/min at week 12; study medications were continued and his eGFR remained stable (50–59 mL/min) thereafter. No subjects discontinued study medications for renal function changes or other adverse events. Darunavir trough concentration were lower on the new regimen than on darunavir/ritonavir 800/100 mg (n = 5; p < 0.05).

**Conclusions:**

Despite low darunavir trough concentrations, treatment simplification to a two-pill, once-daily regimen of E/C/F/TDF plus darunavir was safe and effective for 48 weeks among 10 selected treatment-experienced HIV-infected patients.

*Trial registration* The study protocol was registered with ClinicalTrials.gov (NCT02199613) on July 22, 2014

## Background

Despite the success of current highly active antiretroviral therapy regimens, some HIV-infected patients require regimens encompassing multiple drug classes because of transmitted or acquired drug-resistant virus [1, 2]. Such regimens usually include a ritonavir-boosted protease inhibitor and generally comprise several pills in two or more daily doses, making adherence a challenge for many patients [3]. For example, the TRIO regimen (raltegravir 400 mg twice daily, etravirine 200 mg twice daily, and darunavir/ritonavir 600 mg/100 mg twice daily), which is effective and often used in patients harboring multi-drug resistant HIV, comprises six pills twice daily, and more if nucleoside reverse transciptase inhibitors (NRTIs) are taken as well (as was the case in the majority of subjects in the ANRS 139 TRIO study) [4]. Over the longer term, high pill burden is a major factor contributing to treatment fatigue among HIV-infected patients prescribed antiretroviral therapy, with important consequences including medication nonadherence and treatment failure [5].

In recent years, first-line regimens have included the use of a fixed-dose once-daily combination tablet consisting of the integrase inhibitor elvitegravir 150 mg (E) with a pharmacologic boosting agent (cobicistat 150 mg [C]) and two reverse transcriptase inhibitors (a nucleoside: emtricitabine or FTC 200 mg [F] and a nucleotide: tenofovir disoproxil fumarate 300 mg [TDF]) [6]. Cobicistat has been shown to also adequately boost plasma levels of protease inhibitors including darunavir [7]. In addition, there is evidence that once-daily boosted darunavir 800 mg is as effective as the twice-daily boosted darunavir 600 mg in treatment-experienced patients in the absence of darunavir resistance-associated mutations [8]. Pharmacokinetic studies support the use of once daily darunavir 800 mg in this population: 24-h post-dose minimum plasma concentrations of darunavir (when given with ritonavir 100 mg) remain above 55 ng/mL, the half maximal effective concentration (EC_50_) for wild-type (non-protease inhibitor-resistant) virus [9]. The elvitegravir/cobicistat/emtricitabine/tenofovir DF (E/C/F/TDF) fixed-dose formulation may allow construction of a two-pill once-daily salvage regimen containing an integrase inhibitor, two nucleoside/nucleotides, and a boosted protease inhibitor: E/C/F/TDF and darunavir 800 mg. Since both E/C/F/TDF and darunavir are recommended to be taken once a day with food [6, 10], this constitutes a truly compact once-daily multi-class regimen.

Some early pharmacokinetic (PK) studies suggested that steady-state trough concentrations (C_trough,ss_) of both darunavir and elvitegravir may be lower when E/C/F/TDF and darunavir are given together, compared to levels obtained when elvitegravir/cobicistat and darunavir (boosted either with cobicistat or ritonavir) are given separately [11, 12]. However, the clinical implications of a potential decrease in C_trough,ss_ are unclear, particularly given the potency of boosted protease-inhibitor-based regimens. Given the potential benefits of treatment simplification in patients receiving complex salvage regimens, we undertook to evaluate the use of once-daily E/C/F/TDF with darunavir as a simplification strategy for treatment-experienced patients who had already achieved virologic suppression on a multi-drug, multi-class antiretroviral regimen, with one-time PK testing and longitudinal viral load monitoring. In view of the known potential for TDF to cause nephrotoxicity [13–15], we also monitored renal function and other safety parameters.

## Methods

### Study design

We conducted a single arm, open-label study of regimen simplification to E/C/F/TDF plus darunavir 800 mg daily from stable therapy including two nucleoside/nucleotide reverse transcriptase inhibitors, a ritonavir-boosted protease inhibitor (atazanavir or darunavir), and raltegravir or dolutegravir.

### Study population

The study enrolled HIV positive adults (≥ 19 years of age) with plasma viral load consistently < 200 copies/mL for ≥ 6 months. Subjects were excluded if they had prior documented virologic rebound > 1000 copies/mL on an integrase inhibitor-containing regimen; had evidence on any previous genotypic testing of resistance mutations which would compromise activity of elvitegravir, darunavir, or tenofovir; were currently receiving any nonnucleoside reverse transcriptase inhibitor (NNRTI); were pregnant or breast-feeding; or had any contraindications to tenofovir DF, emtricitabine, elvitegravir, or cobicistat (e.g. previous significant toxicity, intolerance, or were receiving medications with significant drug interactions with the study drugs). The eligibility criteria included having estimated glomerular filtration rate (eGFR) ≥ 70 mL/min, as recommended by the manufacturer of E/C/F/TDF [6]; however, waivers were allowed for participants with a stable eGFR ≥ 60 mL/min, based on available evidence indicating the safety of E/C/F/TDF in HIV-infected patients with this degree of mild renal impairment [16].

### Determination of plasma concentration of study drugs

A plasma sample for measurement of darunavir C_trough,ss_ (pre-dose) was collected at baseline before the switch in subjects receiving once-daily darunavir in their pre-switch regimen.

All subjects took study medication (E/C/F/TDF and darunavir) with food under observation in the clinic on Day 14 or later after starting the new regimen. Plasma samples for drug level testing were drawn immediately pre-dose (C_trough,ss_) and at 1, 2, 3, 4, 5, 6, and 8 h post-dose, then once on the following day for a 24-h post-dose C_trough,ss_. Plasma samples were frozen and stored at – 80 °C until analysis.

Darunavir in stored plasma samples was measured in the BC Centre for Excellence in HIV/AIDS Laboratory using a fully validated method [17]. Addition of elvitegravir and cobicistat to the method was partly validated, including selectivity, linearity, accuracy and recovery, inter- and intra-run repeatability, and stability. External controls were included for darunavir and elvitegravir, but were not available for cobicistat. Lower limits of quantification were 70 ng/mL for darunavir, 80 ng/mL for elvitegravir, and 50 ng/mL for cobicistat. Plasma tenofovir and emtricitabine levels were not measured.

### Safety and efficacy assessments

Medical history and physical exam were conducted at baseline, and clinical adverse event assessment and medication update performed at weeks 2, 12, 24, 36, and 48. HIV plasma viral load (COBAS Ampliprep Taqman HIV-1 assay, Roche Diagnostics Systems, Laval, Quebec, Canada) was measured at baseline, at weeks 2 and 4, then every 4 weeks throughout the study. The following laboratory assessments were conducted at baseline and at weeks 12, 24, 36, and 48: CD4 cell counts (absolute and fraction), CD4/CD8 ratio, renal function (serum creatinine, eGFR, serum phosphorus, urinalysis, urine albumin to creatinine ratio [UACR]), AST, ALT, total bilirubin, fasting blood sugar, fasting lipid parameters (total cholesterol, LDL, HDL, total cholesterol/HDL, triglycerides, and apolipoprotein B [apoB]), high-sensitivity C-reactive protein (hsCRP), and pregnancy test for women of child-bearing potential .

### Study endpoints and statistical analyses

The primary endpoint was the proportion of subjects with plasma viral load < 200 copies/mL at week 12 following the regimen switch, with secondary endpoints at weeks 24 and 48. The threshold of 200 copies/mL was chosen based on data showing that low level viremia between 50–199 copies/mL is not associated with virological failure or clinical outcomes [18], and is consistent with the definition of virologic failure in international antiretroviral treatment guidelines [19, 20]. Changes in CD4 cell count (absolute and fraction), CD4/CD8 ratios, creatinine, eGFR, serum phosphorus, ALT, AST, total bilirubin, fasting glucose and lipid parameters, and hsCRP between baseline and week 48 were determined using a Wilcoxon signed rank sum test with significance level 0.05. For subjects receiving once daily darunavir prior to switching to the study regimen, darunavir C_trough,ss_ at baseline and day 14 were compared using Wilcoxon signed rank sum test. Elvitegravir and cobicistat concentrations were compared to historical controls.

No sample size calculation was performed for this study. A convenience sample of ten patients was enrolled.

## Results

### Baseline characteristics

Ten HIV-infected subjects, 8 men and 2 women, were enrolled and started study medications between October 2014 and February 2016 (Table 1). Median age was 50.5 years (range 33–71), weight was 87.5 kg (range 56–101.5), and CD4 cell count was 505 cells/mm^3^ (range 50−1020). All had viral load < 40 copies/mL except one whose viral load was 134 copies/mL, considered to be within the limits of error of the assay [21]. Median eGFR at baseline was 81 mL/min (range 60–102). The two subjects who entered the study with eGFR < 70 mL/min had stable renal function, with mildly decreased eGFR between 60 and 70 mL/min for at least 6 months prior to the study baseline visit. All study subjects were receiving tenofovir DF and emtricitabine; 9 were receiving raltegravir and one dolutegravir; 7 were receiving ritonavir-boosted darunavir (6 once daily and one twice daily) and 3 were receiving ritonavir-boosted atazanavir. No subjects were receiving any concomitant medications that would be expected to affect the plasma levels of the antiretrovirals.

**Table 1:** Baseline characteristics of study participants (n = 10)

Subject no.	Gender	Age, years	Weight, kg	eGFR, mL/min	Viral load, copies/mL	CD4, cells/mm^3^	Antiretroviral regimen (with tenofovir DF and emtricitabine)
1	Male	59	92.5	78	< 40	180	RAL 400 mg twice daily DRV 800 mg daily Ritonavir 100 mg daily
2	Male	47	89.5	60	< 40	510	RAL 400 mg twice daily DRV 800 mg daily Ritonavir 100 mg daily
3	Male	71	63	62	< 40	400	RAL 400 mg twice daily ATV 300 mg daily Ritonavir 100 mg daily
4	Male	55	101.5	85	< 40	900	RAL 400 mg twice daily DRV 800 mg daily Ritonavir 100 mg daily
5	Female	42	56	84	< 40	900	RAL 400 mg twice daily DRV 600 mg twice daily Ritonavir 100 mg twice daily
6	Male	33	85.5	102	< 40	410	RAL 400 mg twice daily ATV 300 mg daily Ritonavir 100 mg daily
7	Male	53	72	95	< 40	500	RAL 400 mg twice daily DRV 800 mg daily Ritonavir 100 mg daily
8	Male	56	95.5	74	< 40	700	DTG 50 mg daily DRV 800 mg daily Ritonavir 100 mg daily
9	Female	48	84	100	< 40	50	RAL 400 mg twice daily DRV 800 mg daily Ritonavir 100 mg daily
10	Male	47	98.5	75	134	1020	RAL 400 mg twice daily ATV 300 mg daily Ritonavir 100 mg daily

*eGFR* estimated glomerular filtration rate, *RAL* raltegravir, *DTG* dolutegravir, *DRV* darunavir, *ATV* atazanavir

### Archived antiretroviral drug resistance mutations

Nine subjects had evidence of archived drug-resistant virus on previous genotypic testing, all of whom had M184V/I (conferring resistance to lamivudine and emtricitabine) (Table 2). Five subjects had had thymidine analogue mutations including 41L and 215Y or F, associated with reduced susceptibility to tenofovir DF: enrolment in the study was judged to be safe for these subjects because the other study drugs (darunavir and elvitegravir) were fully active. Six subjects had resistance to NNRTIs, and four had protease-inhibitor-associated mutations, but retained susceptibility to darunavir.

**Table 2:** Archived antiretroviral drug resistance mutations among study participants

Subject no.	Major NRTI resistance mutations		Major NNRTI resistance mutations	Major PI resistance mutations
	Non-TAMs	TAMs		
1	184V	67N, 70R, 219Q	103N	ND
2	184V, 74V	67N, 70R, 219Q	181C, 190A	ND
3	184V	41L, 210W, 215Y	ND	ND
4	184V	70R	ND	ND
5	184V, 74I	41L, 67N, 70R, 215F, 219Q	103N	84V, 90M
6	184V	41L, 67N, 70R, 215Y, 219Q	103N, 181C, 190S	30N, 88D
7	184V	41L, 215Y	103N	54V, 82A
8	184V	41L, 215Y	98G^a^	46L
9	184I, 70E	ND	ND	ND
10	ND	ND	ND	ND

Reference: Stanford HIV Drug Resistance Database. Major HIV-1 Drug Resistance Mutations, Updated summary March 9, 2015. http://hivdb.stanford.edu

*NRTI* nucleoside analogue reverse transcriptase inhibitor, *TAMs* thymidine analogue mutations, *NNRTI* nonnucleoside analogue reverse transcriptase inhibitor, *PI* protease inhibitor, *ND* none detected

^a^Although not listed as major NNRTI mutation, 98G confers resistance to nevirapine

### Efficacy results

**Fig. 1:**
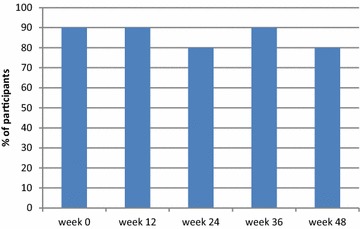
Proportion of participants (n = 10) with plasma viral load < 40 copies/mL

**Fig. 2:**
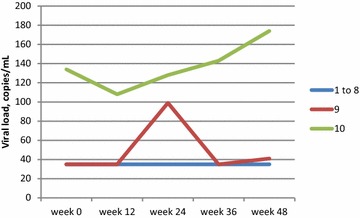
Viral load of each study participant

**Fig. 3:**
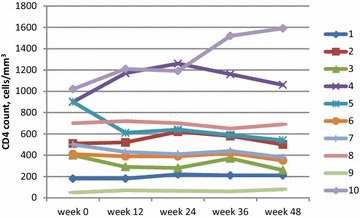
CD4 cell count of each study participant

All 10 subjects had plasma viral load < 200 copies/mL at baseline and at every time point during the study. Nine subjects had viral load < 40 copies/mL at baseline, and viral load remained < 40 copies/mL in 9 at week 12, in 8 at week 24, and in 8 at week 48 (Fig. 1); the subject whose viral load was detectable at week 48 had a viral load of 41 copies/mL. Subject 10’s viral load remained detectable at < 200 copies/mL at each time point during the study, and was 174 copies/mL at week 48 (Fig. 2). Among all 10 subjects, no significant changes were observed between baseline and week 48 in absolute CD4 cell count (median 505 and 440 cells/mm^3^, respectively) (Fig. 3), CD4 fraction (median 25 and 26%, respectively), or CD4/CD8 ratio (median 0.61 and 0.63, respectively) (p > 0.05 for all).

### Safety results

In the group as a whole, no significant changes were observed between baseline and week 48 in creatinine, eGFR, serum phosphorus, ALT, AST, total bilirubin, glucose, lipid parameters, or hsCRP (p > 0.05 for all). No subjects discontinued study medications during the 48-week study for renal function changes or other adverse events. Subject three experienced a decrease in eGFR from 62 mL/min at baseline to 52 mL/min at week 12; study medications were continued and his eGFR remained stable (50–59 mL/min) thereafter. At study entry, this patient had been receiving a tenofovir DF-containing regimen for 29 months and had hypophosphatemia (serum phosphorus 0.66 mmol/L, lower limit of normal 0.80 mmol/L) and proteinuria (UACR 19.2 mg/mmol, upper limit of normal 2.0 mg/mmol), which persisted over the course of the study; at week 48, his serum phosphorus was 0.72 mmol/L and UACR was 33.9 mg/mmol. After week 48, study medications were discontinued and replaced with abacavir, lamivudine, raltegravir, and darunavir/ritonavir; 3 months later, his eGFR was 63 mL/min, serum phosphorus was 0.92 mmol/L, and UACR was 2.3 mg/mmol. No significant clinical or laboratory adverse events were observed in any other study subjects.

### Pharmacokinetics of darunavir, elvitegravir, and cobicistat

#### Darunavir levels

Six subjects were receiving darunavir/ ritonavir once daily prior to study entry, of whom 5 had 24-h post-dose darunavir C_trough,ss_ measured at both baseline and 2 weeks after the switch to E/C/F/TDF and darunavir (the other subject [number 7 in Tables 1 and 2] had taken his medications before the study baseline visit so a pre-dose sample could not be drawn). The median darunavir C_trough,ss_ for these 5 subjects decreased from 981 ng/mL (range 667–1150) at baseline to 431 ng/mL (range 96–784) at week 2 (p ≤ 0.05). Among all nine subjects who had plasma drug levels measured after the switch to E/C/F/TDF and darunavir (median 14 days, range 14–28 days after the switch; Subject 6 was not available for week 2 sampling), the median darunavir C_trough,ss_ was 482 ng/mL (range 96–848).

Mean and median darunavir C_trough,ss_ are shown in Table 3 for the purpose of comparison with data from the literature. The darunavir C_trough,ss_ we observed with ritonavir prior to the switch to E/C/F/TDF tended to be lower than C_trough,ss_ published in the literature for darunavir/ritonavir 800 mg/100 mg once daily [22, 23]. After the switch to E/C/F/TDF, the darunavir C_trough,ss_ we observed were higher than the extrapolated C_trough,ss_ reported in the presence of E/C/F/TDF by Ricard et al [12], but lower than the measured 24-h darunavir C_trough,ss_ with the same regimen reported by Gutierrez-Valencia et al [24].

**Table 3:** Plasma concentrations of darunavir and elvitegravir at 24 h post-dose

Study intervention	N	Darunavir C_trough,ss_, ng/mL		Elvitegravir C_trough,ss_, ng/mL		Refs.
		Median/mean	Range	Median/mean	Range	
DRV/rtv, pre-switch	5	981 (median) 932 (mean)	667–1150	– –	–	
E/C/F/TDF + DRV	9	482 (median) 490 (mean)	96–848	184 (median) 200 (mean)	< 80–435	
DRV/rtv 800/100 mg	335^a^	2041 (median)	368–7242	–	–	[22]
	119	1820 (mean)	IQR 1470–2460	–	–	[23]
DRV/cobi 800/150 mg	298^a^	2150 (mean)	SD 1320	–	–	[25]
	59	1311 (mean)	SD 969	–	–	[25]
	32	1319 (mean)	288–3641	–	–	[24]
E/C/F/TDF (no DRV)	419^a^	–	–	451 (mean)	58–2341	[26]
	32	–	–	250 (mean)	30–762	[24]
E/C/F/TDF + DRV	24	1294 (mean)	163–3641	234 (mean)	92–432	[24]
	8	273 (median)^b^	164–501 (IQR)^b^	–	–	[12]
E/C/F/TAF + DRV	15	1250 (mean)	NA	464 (mean)	NA	[36]

*C*_*trough,ss*_ trough plasma concentration at steady-state, *DRV* darunavir, *rtv* ritonavir, *cobi* cobicistat, *E/C/F/TDF* elvitegravir/cobicistat/emtricitabine/tenofovir disproxil fumarate, *E/C/F/TAF* elvitegravir/cobicistat/ emtricitabine/ tenofovir alafenamide, *IQR* interquartile range, *SD* standard deviation, *NA* not available

^a^Population pharmacokinetic estimate; ^b^ estimated

The maximum observed darunavir level at 2 weeks was 5840 ng/mL (median), range 3590–7840 ng/mL (n = 9), and was reached at a median of 2.5 h (range 1–5 h) after dosing. This is generally similar to published data for 60 subjects receiving darunavir 800 mg/cobicistat 150 mg with two nucleoside/nucleotide reverse transcriptase inhibitors: mean darunavir C_max_ 7663 ng/mL (standard deviation 1920), median t_max_ 3.5 h (interquartile range [IQR] 2.5–4.3 h) [25].

#### Elvitegravir and cobicistat levels

Among the 9 subjects who had drug levels measured 2 weeks after switching to E/C/F/TDF and darunavir, the median elvitegravir C_trough,ss_ was 184 ng/mL (range < 80–296).

Mean and median elvitegravir C_trough,ss_ are shown in Table 3 for the purpose of comparison with data from the literature. Our observed elvitegravir C_trough,ss_ were somewhat lower than the manufacturer’s population pharmacokinetic estimates for E/C/F/TDF without darunavir [26], but generally similar to 24-h C_trough,ss_ measured by Gutierrez-Valencia et al. among patients taking E/C/F/TDF either with or without darunavir [24].

The maximum observed elvitegravir level was 1230 ng/mL (median), range 651–2400 ng/mL (n = 9), and was reached at a median of 3 h (range 1–8 h) after dosing. In comparison, population PK data for E/C/F/TDF (n = 419) provide a mean C_max_ of 1731 ng/mL (standard deviation 23) with a t_max_ of 4.0 h [6, 26].

Cobicistat levels at 24 h post-dosing were < 50 ng/mL in all 9 of our study subjects with available drug levels. The maximum observed cobicistat level was 769 ng/mL (median), range 493–1090 ng/mL (n = 9), and was reached at a median of 2 h (range 2–4 h) after dosing. In published data for 60 subjects receiving darunavir 800 mg/cobicistat 150 mg with two nucleoside/nucleotide reverse transcriptase inhibitors, mean cobicistat C_0h_ was 76 ng/mL (standard deviation 186), mean C_max_ was 991 ng/mL (standard deviation 331), and median t_max_ was 3.5 h (IQR 2.0–4.5) [25].

## Discussion

Among 10 treatment-experienced HIV-infected patients who had viral load consistently < 200 copies/mL on a multiple-class antiretroviral regimen, a switch to a two-pill once-daily regimen of E/C/F/TDF and darunavir was safe and effective in maintaining virologic suppression for 48 weeks. One patient had pre-existing TDF-related renal tubular toxicity which persisted but did not worsen on the study regimen, and improved when he was changed to a non-TDF-containing regimen after completion of the study.

Among the 5 patients receiving once-daily darunavir/ritonavir at baseline, darunavir C_trough,ss_ levels were significantly lower after the switch to E/C/F/TDF plus darunavir (the median decreased from 981 to 431 ng/mL), and darunavir C_trough,ss_ levels were low for all 9 subjects with drug levels available on E/C/F/TDF plus darunavir (median 482 ng/mL). Previous studies have shown darunavir C_trough,ss_ to be lower when boosted with cobicistat than with ritonavir, both in healthy volunteers (21–24% lower with cobicistat) [27] and HIV-infected patients (30% lower with cobicistat) [24]. However, the magnitude of the effect we observed (> 50% reduction in darunavir C_trough,ss_) was greater than that observed in the previous studies. This is particularly striking since our subjects’ darunavir C_trough,ss_ levels on darunavir /ritonavir (median 981 ng/mL) were already more than 50% lower than those reported in the literature for HIV-infected patients receiving darunavir /ritonavir [22, 23]. The reason for this is unclear, as drug–drug interactions are not expected between darunavir and either raltegravir or dolutegravir (taken concomitantly by 4 subjects and 1 subject, respectively) [28–30]. Possibly as a result of these low baseline levels, our patients’ darunavir C_trough,ss_ levels on E/C/F/TDF plus darunavir were less than 40% of those expected with darunavir /cobicistat without elvitegravir (darunavir C_trough,ss_ approximately 1300 ng/mL) [24, 25]. The low darunavir C_trough,ss_ seen in our patients on E/C/F/TDF plus darunavir support an earlier retrospective study that used extrapolated levels and suggested a significant drug–drug interaction between darunavir and E/C/F/TDF [12]; however, a subsequent larger study showed darunavir C_trough,ss_ were similar in 24 patients receiving E/C/F/TDF plus darunavir as in 32 patients receiving darunavir /cobicistat without elvitegravir [24]. Although our study is small, we were able to prospectively measure darunavir C_trough,ss_ in the same patients before and after the switch from darunavir/ritonavir to E/C/F/TDF plus darunavir, and to demonstrate a statistically significant decrease in darunavir C_trough,ss_ after the switch. On the other hand, while the observed darunavir concentrations were low, they remained 2- to 15-fold higher than the protein-adjusted 50% inhibitory concentration (IC_50_) for darunavir against wild-type virus (55 ng/mL) [31], and the study regimen maintained antiviral efficacy throughout the 48 week study in all 10 patients. As an alternative boosted protease inhibitor-elvitegravir combination regimen, atazanavir would not be an ideal option; coadministration with elvitegravir/cobicistat has been shown to result in significant lowering of atazanavir trough levels [32].

The elvitegravir C_trough,ss_ in our patients receiving E/C/F/TDF plus darunavir (median elvitegravir C_trough,ss_ 184 ng/mL, mean 200 ng/mL) were about 40% of those reported by the manufacturer for E/C/F/TDF without darunavir based on population PK analysis (mean 451 ng/mL) [26]. This is despite the fact that our patients were instructed to take their medications with food, and were observed to do so on the day prior to the 24-h post-dose draw, as recommended to optimize elvitegravir exposure from the coformulation [6, 33]. However, the elvitegravir concentrations we observed were similar those observed by Gutierrez-Valencia et al. in patients receiving E/C/F/TDF, either with darunavir (mean elvitegravir C_trough,ss_ 234 ng/mL) or without darunavir (mean elvitegravir C_trough,ss_ 250 ng/mL) [24]. Since we do not have elvitegravir levels in our patients in the absence of darunavir, we cannot comment on whether there is a significant drug–drug interaction which lowers elvitegravir C_trough,ss_, but the study by Gutierrez-Valencia et al. suggests that this may not be the case [24]. In 7 of 9 subjects in our study, the observed elvitegravir C_trough,ss_ were more than 2-fold above the protein-adjusted 95% inhibitory concentration (IC_95_) for elvitegravir against wild type virus (45 ng/mL) [34]; the other two patients had elvitegravir C_trough,ss_ of 82 and < 80 ng/mL. Nevertheless, virologic efficacy was maintained in all 10 patients.

Cobicistat C_trough,ss_ were below the lower limit of the assay (< 50 ng/mL) in all cases, and consistent with previous studies utilizing cobicistat as a booster for either elvitegravir or darunavir or both. Tashima et al. reported mean 24-h cobicistat levels of 33 ng/mL (standard deviation 95) among 59 HIV-infected patients taking darunavir/cobicistat with emtricitabine and tenofovir DF [25]. Gutierrez-Valencia et al. reported mean 24-h cobicistat concentrations of 20.2 ng/mL (IQR 11.2–33.1) in patients taking E/C/F/TDF with darunavir, and similar concentrations among those taking E/C/F/TDF without darunavir [24]. While we were unable to quantify cobicistat levels below 50 ng/mL, it appears there was enough cobicistat present to adequately boost elvitegravir, so low cobicistat concentrations are unlikely to explain the low darunavir concentrations seen in our patients taking E/C/F/TDF plus darunavir. In any case, cobicistat C_trough,ss_ is probably less important than area under the plasma concentration-time curve (AUC) in terms of its pharmacological boosting properties [35].

Limitations of our study include the small sample size, non-randomized design, and the lack of full validation for the elvitegravir and cobicistat assays, although the darunavir assay was fully validated. Also our study was conducted with the older E/C/F/TDF formulation. Further investigation of this approach may be warranted, including the new tenofovir alafenamide (TAF) formulation. PK data are available for 15 treatment-experienced HIV patients participating in a switch study to E/C/F/TAF plus darunavir; their mean darunavir C_trough,ss_ was 1250 ng/mL and elvitegravir C_trough,ss_ was 464 ng/mL (no range or IQR available) [36]. There is no reason to expect a substantial difference in the interactions between elvitegravir, cobicistat, and darunavir when coadministered with TAF vs. TDF. The main difference to be expected would be lower plasma tenofovir levels in the presence of darunavir with E/C/F/TAF than with E/C/F/TDF, due to P-glycoprotein induction by darunavir and the resultant decrease in intestinal absorption of TAF (a P-glycoprotein substrate) [10, 37]. Indeed, in the PK substudy of the E/C/F/TAF plus darunavir switch study, plasma concentrations of TAF were at the lower end of the efficacious range, and plasma tenofovir exposure was “markedly lower” than that observed with E/C/F/TDF in previous studies [36]. Nevertheless, simplification to E/C/F/TAF plus darunavir was shown to be safe and efficacious, maintaining virologic suppression (viral load < 50 copies/mL) in 94% of 89 participants in the study [36]. Since we did not measure plasma tenofovir levels in the present study, we are unable to say whether the same effect occurred in our cohort.

## Conclusions

In conclusion, E/C/F/TDF plus darunavir was safe and effective as a treatment simplification option for 10 selected treatment-experienced HIV-infected patients. Although darunavir C_trough,ss_ with E/C/F/TDF plus darunavir were lower than with ritonavir-boosted darunavir in this small study, virologic suppression was maintained in all subjects for 48 weeks.

## Abbreviations

apoB: apolipoprotein B
AUC: area under the plasma concentration-time curve
C_0h_: plasma concentration at 0 h
C_max_: maximum plasma concentration
C_trough,ss_: trough plasma concentration at steady state
EC_50_: half maximal effective concentration
E/C/F/TDF: elvitegravir/cobicistat/emtricitabine/tenofovir disproxil fumarate
eGFR: estimated glomerular filtration rate
FTC: emtricitabine
hsCRP: high-sensitivity C-reactive protein
IC_50_: 50% inhibitory concentration
IC_95_: 95% inhibitory concentration
IQR: interquartile range
NNRTI: nonnucleoside reverse transcriptase inhibitor
NRTI: nucleoside reverse transcriptase inhibitor
PK: pharmacokinetics
TAF: tenofovir alafenamide
TDF: tenofovir disoproxil fumarate
t_max_: time to maximum plasma concentration
UACR: urine albumin to creatinine ratio

## References

[1] Harris M, Harrigan PR, Montaner JSG, Volberding PA, Sande MA, Lange J, Greene WC, Gallant JE (2008). Chapter 46: Antiretroviral Therapy of Drug-resistant HIV. Global HIV/AIDS medicine.

[2] Wainberg MA, Zaharatos GJ, Brenner BG (2011). Development of antiretroviral drug resistance. N Engl J Med.

[3] Chesney MA (2000). Factors affecting adherence to antiretroviral therapy. Clin Infect Dis.

[4] Yazdanpanah Y, Fagard C, Descamps D (2009). High rate of virologic suppression with raltegravir plus etravirine and darunavir/ritonavir among treatment-experienced patients infected with multi-drug resistant HIV: results of the ANRS 139 TRIO trial. Clin Infect Dis.

[5] Claborn KR, Meier E, Miller MB, Leffingwell TR (2015). A systematic review of treatment fatigue among HIV-infected patients prescribed antiretroviral therapy. Psychol Health Med..

[6] Gilead Sciences Canada Inc. Stribild Product Monograph. Mississauga, Ontario, Canada; March 7, 2017.

[7] Shah BM, Schafer JJ, Priano J, Squires KE (2013). Cobicistat: a new boost for the treatment of human immunodeficiency virus infection. Pharmacotherapy.

[8] Cahn P, Fourie J, Grinzstejn B (2011). Week 48 analysis of once-daily vs. twice-daily drunavir/ritonavir in treatment-experienced HIV-1-infected patients. AIDS.

[9] Boffito M, Miralles D, Hill A (2008). Pharmacokinetics, efficacy, and safety of darunavir/ritonavir 800/100 mg once-daily in treatment-naïve and -experienced patients. HIV Clin Trials.

[10] Janssen Inc. Prezista Product monograph. Toronto, Ontario, Canada; April 7, 2017.

[11] Ramanathan S, Wang H, Szwarcberg J, Kearney BP. Safety/tolerability, pharmacokinetics and boosting of twice-daily cobicistat administered alone or in combination with darunavir or tipranavir. In: 13th International Workshop on Clinical Pharmacology of HIV Therapy. Barcelona, Spain. April 16-18, 2012. (poster P08).

[12] Ricard F, Wong A, Lebouche B, et al. Low darunavir concentrations in patients receiving Stribild (elvitegravir/cobicistat/emtricitabine/tenofovir disproxil fumarate) and darunavir once daily. In: 16th International Workshop on Clinical Pharmacology of HIV and Hepatitis Therapy. May 26–28, 2015. Washington, DC, USA. (poster 50).

[13] Ryom L, Mocroft A, Kirk O (2013). Association between antiretroviral exposure and renal impairment among HIV-positive persons with normal baseline renal function: the D: a: D study. J Infect Dis.

[14] Islam FM, Wu J, Jansson J, Wilson DP (2012). Relative risk of renal disease among people living with HIV: a systematic review and meta-analysis. BMC Public Health.

[15] Scherzer R, Estrella M, Li Y, Deeks SG, Grunfeld C, Shlipak MG (2012). Association of tenofovir exposure with kidney disease risk in HIV infection. AIDS.

[16] Post FA, Winston J, Andrade-Villanueva JF (2015). Elvitegravir/cobicistat/emtricitabine/ tenofovir DF in HIV-infected patients with mild-to-moderate renal impairment. J Acquir Immune Defic Syndr.

[17] Gonzalez-Serna A, Swenson LC, Watson B, Auyeung K, Montaner J, Harrigan R (2016). A single untimed plasma drug concentration measurement during low-level HIV viremia predicts virologic failure. Clin Microbiol Infection.

[18] The Antiretroviral Therapy Cohort Collaboration (2015). (ART-CC). Impact of low-level viremia on clinical and virological outcomes in treated HIV-1-infected patients. AIDS.

[19] Panel on antiretroviral guidelines for adults and adolescents. Guideleins for the use of antiretroviral agents in HIV-1-infected adults and adolescents. Department of Health and Human Services. http://www.aidsinfo.nih.gov/ContentFiles/AdultandAdolescentGL.pdf. Accessed 13 Oct 2017.

[20] Günthard HF, Saag MS, Benson CA (2016). Antiretroviral drugs for treatment and prevention of HIV infection in adults: 2016 recommendations of the international antiviral society-USA Panel. JAMA. J Am Med Assoc.

[21] Lima V, Harrigan R, Montaner JS (2009). Increased reporting of detectable plasma HIV-1 RNA levels at the critical threshold of 50 copies per milliliter with the Taqman assay in comparison to the Amplicor assay. J Acquir Immune Defic Syndr.

[22] Sekar V, Vanden Abeele C, Van Baelen B, et al. Pharmacokinetic/pharmacodynamic analyses of once-daily darunavir in the ARTEMIS study. 15th Conference on Retroviruses and Opportunistic Infections. February 3–6, 2008. Boston, USA. (poster 769).

[23] Gutierrez-Valencia A, Torres-Cornejo A, BenMarzouk-Hidalgo OJ (2014). Darunavir minimum plasma concentration and ritonavir-boosted darunavir monotherapy outcome in HIV-infected patients. Antivir Ther.

[24] Gutierrez-Valencia A, Benmarzouk-Hidalgo OJ, Llaves S (2017). Pharmacokinetic interactions between cobicistat-boosted elvitegravir and darunavir in HIV-infected patients. J Antimicrob Chemother.

[25] Tashima K, Crofoot G, Tomaka FL (2014). Cobicistat-boosted darunavir in HIV-1-infected adults: week 48 results of a Phase IIIb, open-label single-arm trial. AIDS Res Ther.

[26] Ramanathan S, Wei X, Szwarcberg J, Chang S, Cheng AK, Kearney BP. Exposure-response analysis of once-daily elvitegravir administered as EVG/COBI/FTC/TDF single tablet regimen (Quad STR) in HIV-1 infected patients. In: 19th Conference on Retroviruses and Opportunistic Infections. March 5–8, 2012. Seattle, USA. (poster 622).

[27] Kakuda TN, Opsomer M, Timmers M (2014). Pharmacokinetics of darunavir in fixed-dose combination with cobicistat compared with coadministration of darunavir and ritonavir as single agents in healthy volunteers. J Clin Pharm..

[28] Jackson A, Watson V, Back D (2011). Plasma and Intracellular Pharmacokinetics of darunavir/ritonavir once daily and raltegravir once and twice daily in HIV-1 infected individuals. J Acquir Immune Defic Syndr.

[29] Garvey L, Latc N, Erlwein OW (2010). The effects of a nucleoside-sparing antiretroviral regimen on the pharmacokinetics of ritonavir-boosted darunavir in HIV type-1-infected patients. Antivir Ther.

[30] Song I, Min SS, Borland J (2011). The effect of lopinavir/ritonavir and darunavir/ritonavir on the HIV integrase inhibitor S/GSK1349572 in healthy participants. J Clin Pharmacol.

[31] De Meyer S, Azijn H, Surleraux D (2005). TMC114, a novel human immunodeficiency virus type 1 protease inhibitor active against protease inhibitor-resistant viruses, including a broad range of clinical isolates. Antimicrob Agents Chemother.

[32] S Ramanathan, H Wang, T Stondell, A Cheng, and BP Kearney. Pharmacokinetics and Drug Interaction Profile of Cobicistat boosted-Elvitegravir with Atazanavir, Rosuvastatin or Rifabutin. In: 13th International Workshop on Clinical Pharmacology of HIV Therapy, Barcelona, Spain. April 16–18, 2012.

[33] Cattaneo D, Baldelli S, Minisci D (2016). When food can make the difference: the case of elvitegravir-based co-formulation. Int J Pharm.

[34] Ramanathan S, Mathias AA, German P, Kearney BP (2011). Clinical pharmacokinetic and pharmacodynamic profile of the HIV integrase inhibitor elvitegravir. Clin Pharmacokinet.

[35] Barcelo C, Gaspar F, Aouri M (2016). Population pharmacokinetic analysis of elvitegravir and cobicistat in HIV-1-infected individuals. J Antimicrob Chemother.

[36] Huhn GD, Tebas P, Gallant J (2017). A randomized, open-label trial to evaluate switching to elvitegravir/cobicistat/emtricitabine/tenofovir alafenamide plus darunavir in treatment-experienced HIV-1-infected adults. J Acquir Immune Defic Syndr.

[37] Gilead Sciences Canada Inc. Genvoya Product Monograph. Mississauga, Ontario, Canada; May 24, 2017.

